# Poster 1002: Galectin-3 as predictive biomarker of airways remodeling modulation in omalizumab treated severe asthma patients

**DOI:** 10.1186/1939-4551-7-S1-P3

**Published:** 2014-02-03

**Authors:** Pierluigi Mauri, Annamaria Riccio, Rossana Rossi, Dario Di Silvestri, Louise Benazzi, Laura De Ferrari, Roberto W Dal Negro, G Walter Canonica

**Affiliations:** 1 OMICS Lab CNR, Milano, Italy; 2 Genoa University, Dept Of Internal Medicine Allergy and Respiratory Diseases, Genoa, Italy; 3 CMS Verona, Verona, Italy

## Background

A significant effect of Omalizumab treatment on bronchial remodeling modulation(reduction of Reticular Basal Membrane-RBM- thickening) by means of histological evaluation of bronchial biopsies was documented after therapy (Riccio et al. 2012). But we surprisingly found two groups , Responders-R (reduction of tickening of RBM) and Non Responders-NR (increasing or stable RBM). We then applied the proteomic analysis to the bronchial specimens. We used MudPIT (Multidimensional Protein Identification Technology) proteomic approach, a highthroughput methodology that allows the identification of hundreds/thousands of proteins for a single complex sample, evaluation of differential abundance, characterization of involved molecular pathways and sub-typing diseases (Brambilla et al., 2012).

## Methods

Eight patients were studied (7 non smoker; 3 females; range of age 40-62 years; mean age 47.0±9.7sd;mean BMI 23.8±3.1sd; mean total plasma IgE 309.4 IU/l±218.2sd; mean FEV1 56.2%pred±14.5sd; ACT score 11.1±2.9sd) suffering from severe persistent atopic asthma treated by Omalizumab for 12 months.

The identificationof the proteins (over 800) obtained by MudPIT plus the Hierarchical clustering identified some possible markers, including periostin. The identified proteins were plotted into the interactomic networks by means of Cytoscape software and its plug-in, permitting to identify the main metabolic clusters involved in the NRvs R differentiation.

## Results

The major differences between R and NR were detected in the Extra Cellular Matrix group of proteins. Among them, Galectin-3 (Gal-3), a IgE binding protein. Gal-3 is a regulatory molecule acting at variuos stages from acute to chronic inflammation and tissue fibrogenesis. It was able to define the two groups, being present in all the R patients and absent in NR ones. These data were confirmed by immunochemistry on those same biopsies.

## Conclusions

Galectin 3 can be considered a reliable biomarker to predict modulation of airways remodeling in severe asthma patients treated with omalizumab.

